# Epidemiologic profile and prevalence of live births with orofacial cleft in Brazil: a descriptive study

**DOI:** 10.1590/1984-0462/2024/42/2022234

**Published:** 2023-09-15

**Authors:** Adriana Mendonça da Silva, Rodrigo Tripodi Calumby, Valéria Souza Freitas

**Affiliations:** a Universidade Estadual de Feira de Santana, Feira de Santana, BA, Brazil.

**Keywords:** Epidemiology, Cleft palate, Cleft lip, Congenital abnormalities, Epidemiologia, Fissura palatina, Fenda labial, Anormalidades congênitas

## Abstract

**Objective:**

To describe the epidemiological profile and prevalence of live births with orofacial clefts in Brazil between 1999 and 2020.

**Methods:**

Descriptive study. The population corresponded to live births with isolated orofacial clefts in Brazil registered in the Live Birth Information System between 1999 and 2020. Descriptive variables were selected according to their availability and grouped into socioeconomic and demographic, maternal and child health care, and biological variables. Data were submitted to a descriptive analysis using the Software for Statistics and Data Science (STATA).

**Results:**

During the period, 33,699 children were born with orofacial clefts, and 82.1% (27,677) of them were isolated clefts. Regarding these cases, the majority were cleft lip and palate (9,619 or 34.7%), followed by cleft palate (9,442 or 34.1%), and by cleft lip (8,616 or 31.3%).

**Conclusions:**

Live births with orofacial clefts in Brazil were male, white, with birthweight ≥2,500 g and gestational age ≥37 weeks, born by cesarean section, and with Apgar scores ≥7. The cases were more frequent among mothers who were in their first and single pregnancy and had seven or more prenatal appointments. The mothers were 20 and 29 years old, had eight to ten years of study, and were single. The national prevalence of clefts was 4.24/10,000. The South and Southeast regions of Brazil had the highest prevalence, while the lowest prevalence was recorded in the Northeast and North regions. For the Federative Units, the highest and lowest prevalences were found, respectively, in Paraná and Acre.

## RESUMO

**Objetivo:**

Descrever o perfil epidemiológico e a prevalência dos nascidos vivos com fissuras orofaciais no Brasil entre 1999 e 2020.

**Métodos:**

Estudo descritivo. A população correspondeu aos nascidos vivos com fissuras orofaciais isoladas no Brasil registrados no Sistema de Informação de Nascidos Vivos entre 1999 e 2020. As variáveis descritivas foram selecionadas de acordo com a sua disponibilidade e agrupadas em variáveis socioeconômicas e demográficas, de atenção à saúde materno-infantil e biológicas. Os dados foram submetidos a análise descritiva utilizando o *Software for Statistics and Data Science* (STATA).

**Resultados:**

No período, 33.699 indivíduos nasceram com fissura orofacial no Brasil, e 82,1% (27.677) deles foram fissuras isoladas. Com relação a esses casos, a maioria foi de fissuras de lábio e palato (9.619 ou 34,7%), seguidas por fissura de palato (9.442 ou 34,1%) e por fissura de lábio (8.616 ou 31,1%).

**Conclusões:**

O perfil epidemiológico dos nascidos vivos com fissuras orofaciais no Brasil foi de nascidos do sexo masculino, da raça/cor branca, por parto cesáreo, com peso ao nascer ≥2,500 g, idade gestacional ≥37 semanas e com índices de Apgar ≥7. Os casos foram mais frequentes entre mães que estavam na primeira gestação, única e que haviam realizado sete ou mais consultas de pré-natal. As mães, com maior frequência, tinham entre 20 e 29 anos, apresentavam oito ou mais anos de estudo, eram solteiras e residiam em cidades do interior. A prevalência nacional de fissuras foi de 4,24/10.000. As Regiões Sul e Sudeste apresentaram as maiores prevalências, enquanto as menores foram registradas nas Regiões Nordeste e Norte. Para as Unidades Federativas, as maiores e menores prevalências foram encontradas, respectivamente, no Paraná e no Acre.

## INTRODUCTION

Orofacial clefts (OC) are the most common craniofacial congenital malformation and occur due to a failure in the embryological fusion process.^1^ According to their embryological origin, clefs can be divided as cleft lip with or without palate (CL/P) and cleft palate only (CP). Also, they can be classified based on the presence or absence of any other anomaly such as syndromic (SOC) or non-syndromic (NSOC) clefts, respectively.^2^ The latter can also be named as isolated clefs, since there are no other anomalies or concomitant syndromes.

Worldwide, it is estimated that they affect approximately 1.5 to 1,000 births, which corresponds to about 220,000 new cases per year.^3^ However, this rate can vary greatly from country to country. The highest prevalences were found in Japan (20/10,000), Canada (10.5/10,000), the United States (10.2/10,000) and Australia (9.7/10,000).^4^ In low- and middle-income countries, approximately one in every 730 children are born with OC.^5^ In Brazil, few studies have been carried out on the national prevalence of OC, mainly due to reporting and recording difficulties.^6^ The most recent studies^7,8^ found prevalences near 5.1/10,000 live births for the country.

The impacts caused by OC are related to aesthetic, functional and emotional alterations, which can last for the entire life of the individual — such as facial disfigurement, recurrent infections, social stigma, and speech, hearing and teeth formation disabilities.^1^ In addition, OC represent one of the main causes of morbidity in the world.^1^ Despite the many advances in OC treatment options, this continues to be a serious burden worldwide.^9^ The prevalence of OC, together with the need for a long-term multidisciplinary treatment and the economic impact generated by them, has led the World Health Organization to consider them as a public health problem.^10^

The etiology of OC is considered complex and multifactorial, involving the interaction of genetic, environmental and behavioral factors.^2^ Despite these, socioeconomic inequalities may also be related to OC.^11^ A maternal profile of adverse risk, involving less favored social strata and less accessibility to the health system, indicates that such mothers have more difficulty in accessing prenatal care services, which may favor increased morbidity, infant mortality and delayed diagnoses of these malformations.^12^

Therefore, knowledge of factors such as education, family income status and stressful events during pregnancy can provide clues about the inequalities that need to be addressed by health professionals in order to prevent and control the identified risk factors associated with the occurrence of OC, starting with the application of specific measures to promote health during pregnancy.^13^ For this, epidemiological studies that assess the socioeconomic, cultural and environmental conditions of patients with OC are needed.^5,6^ In Brazil, these studies are mostly locally specific, as they propose to describe this event using local data from a specific city, state or region.^14–16^ Thus, it is essential to study population data in a national context, not only to derive situational knowledge on the OC problem in Brazil, but also to aid the planning of public policies for assistance and prevention.^17^ Therefore, the present study aimed to describe the epidemiological profile and prevalence of live births with isolated OC in Brazil between 1999 and 2020.

## METHOD

This is an observational, descriptive study, conducted using data collected in the Live Birth Information System (SINASC) about children born with OC in Brazil between 1999 and 2020. SINASC is a national open access health information system that makes it possible to monitor the population's health situation through the collection and processing of demographic and epidemiological data on newborns, mothers, prenatal care and delivery.

The study population corresponded to all cases of live births in Brazil registered in SINASC as having isolated OC (no record of other anomalies or concomitant syndromes) between 1999 and 2020. The OC classification adopted by SINASC follows the Tenth Revision of the International Statistical Classification of Diseases and Related Health Problems (CID-10), which classifies clefts into the following groups: Q35 (cleft palate — CP), Q36 (cleft lip — CL) and Q37 (cleft lip and palate — CLP). Live births that were coded in SINASC for CL and CP simultaneously were considered as having CLP. Cases of OC registered in association with any other major or minor defects, syndromes or multiple birth defects were excluded. Likewise, cases recorded as cleft uvula, or atypical or oblique cleft were also excluded.^4^

The selection of descriptive variables was carried out taking into account their availability in the database. After that, the variables were grouped into socioeconomic and demographic, maternal and child health care and biological variables (Table 1). The quantitative variables (maternal age, paternal age, number of previous pregnancies, Apgar 1^st^ and 5^th^ minute and birth weight in grams) were categorized according to the classification of the Brazilian Ministry of Health.^14^

**Table 1:** Description and categorization of the selected descriptive variables.

Variables	Description	Categorization
Biological variables		
Orofacial cleft type	According to CID-10	Cleft lip; Cleft palate; Cleft lip with palate.
Sex of newborn	Biological sex of newborn	Male; Female.
Ethnicity of newborn	Color of newborn as declared by the mother	White; Black; Yellow (Asian); Brown; Indigenous.
Maternal age	Number of complete years of mother at the time of delivery	≤19 years old; 20–29 years old; 30–34 years old; 35–39 years old; ≥40 years old.
Paternal age	Number of complete years of newborn's father	≤19 years old; 20–39 years old; ≥40 years old.
Apgar score 1^st^ minute	Assess newborn's general condition and vitality in the first minute. It is a predictor of the infant's chances of surviving the first year of life. It ranges from 0 to 10. A score of 7 or greater indicates that the neonate is in good to excellent physical condition.	<7; ≥7 (satisfactory).
Apgar score 5^th^ minute	Assess newborn's general condition and vitality in the first 5 minutes. It is a predictor of the infant's chances of surviving the first year of life. It ranges from 0 to 10. A score of 7 or greater indicates that the neonate is in good to excellent physical condition.	<7; ≥7 (satisfactory).
Maternal and child health care variables		
Number of previous pregnancies	Number of previous pregnancies, not including current pregnancy.	None; One; Two or more.
Number of prenatal appointments	Number of prenatal appointments.	None; 1 to 3 appointments; 4 to 6 appointments; 7 or more appointments.
Place of delivery	Place where the birth took place.	Home; Hospital; Other health place; Others.
Type of delivery	How the birth took place.	Cesarean delivery; Vaginal delivery.
Birth weight	Weight in grams taken up to the 5^th^ hour after birth.	<2,500 grams (low weight); ≥2,500 grams (normal weight).
Gestational age	Number of weeks of gestation at the time of birth.	Preterm (<37 weeks); Term (≥37 weeks).
Type of pregnancy	Number of conceptuses per pregnancy.	Single; Twins or more.
Socioeconomic and demographic variables		
Maternal education	Degree of maternal education in years of study completed.	<1 year; 1 to 3 years; 4 to 7 years; 8 or more years.
Maternal civil status		Married; Divorced; Single; Consensual union; Widow.

CID-10: Tenth Revision of the International Statistical Classification of Diseases and Related Health Problems.

Data were submitted to statistical analysis using the Software for Statistics and Data Science (STATA), version 10. Descriptive analysis was performed according to socioeconomic, maternal and child care and biological variables in absolute and relative frequencies. Differences between groups were tested using the chi-square test, establishing a significance level of p<0.05. Data were also analyzed in a bivariate way to show the crude association between the sex and the NSOC types occurrence. The prevalence of OC was calculated at the national, regional and federal levels. For this calculation, the number of live births with OC according to the mother's state of residence was divided by the total number of live births in the same year and place and multiplied by 10,000.

This study waived the need for consideration by the Ethics Committee for Research on Human Beings (CEP), considering that the data used are from an open access Brazilian information system, available on the website of the IT Department of the Unified Health System (DATASUS) (http://www.datasus.gov.br), in which the data are presented without identifying the subjects.

## RESULTS

Between 1999 and 2020, there were 65,277,959 live births registered in SINASC. Of these, 33,699 were born with OC, occurring in a proportion of 17.8% (6,022) cases of OC associated with other congenital malformations, and 82.1% (27,677) with isolated OC. Regarding the cases of isolated OC, the majority were CLP (9,619 or 34.7%), followed by CP (9,442 or 34.1%), and by CL (8,616 or 31.1%). The categorical descriptive analysis of these NSOC cases, according to biological, maternal and child health care and socioeconomic/demographic characteristics, can be seen in Tables 2 and 3. In a quantitative analysis, the maternal and paternal mean age were 26 (±6.99) and 31 (±7.79) years, respectively, and the children's mean weight at birth was 2,933 kg (±715.5). The bivariate analysis showed a statistic significant crude association between the sex and the NSOC types occurrence (Table 4).

**Table 2:** Number of live births with orofacial cleft according to biological and socioeconomic characteristics in Brazil between 1999 and 2020

		Total (n)	Total (%)
Type of orofacial cleft			
	Cleft lip	8616	31.1
	Cleft lip with palate	9619	34.7
	Total	27,677	100.0
Sex of newborn			
	Female	11,380	41.1
	Male	16,276	58.8
	Total	27,656	100.0
Ethnicity			
	White	13,847	52.4
	Black	907	3.4
	Asian	102	0.3
	Brown	11,329	42.9
	Indigenous	206	0.7
	Total	26,391	100.0
Maternal age (years old)			
	≤19	4975	17.9
	20–29	13,469	48.6
	30–34	4975	17.9
	35–39	3106	11.2
	40 years or more	1137	4.1
	Total	27,662	100.0
Paternal age (years old)			
	≤19	234	4.4
	20–39	4301	81.9
	40 years or more	715	13.6
	Total	5250	100.0
1^st^ minute Apgar score			
	≥7	21,632	96.7
	<7	729	3.2
	Total	22,361	100.0
5^th^ minute Apgar score			
	≥7	24,797	97.0
	<7	742	2.9
	Total	25,539	100.0
Maternal civil status			
	Single	12,673	4.3
	Married	9910	36.2
	Consensual union	4332	15.8
	Divorced	333	1.2
	Widow	72	0.2
	Total	27,320	100.0
Maternal educational level (years)			
	None	479	1.76
	1 to 3	2063	7.57
	4 to 7	7656	28.1
	8 to 11	12,713	46.6
	12 years or more	4336	15.9
	Total	27,247	100.0

**Table 3:** Number of live births with orofacial cleft according to maternal and child health care characteristics in Brazil between 1999 and 2020.

		Total (n)	Total (%)
Number of previous pregnancies			
	None	9301	37.6
	One	5450	22.0
	Two or more	9943	40.2
	Total	24,694	100.0
Number of prenatal appointments			
	None	681	2.4
	1 to 3	2275	8.3
	4 to 6	7814	28.6
	7 or more	16,542	60.5
	Total	27,312	100.0
Place of delivery			
	Hospital	27,203	98.3
	Home	183	0.6
	Others	55	0.2
	Other health place	230	0.8
	Total	27,671	100.0
Type of delivery			
	Vaginal	12,812	46.4
	Cesarean	14,814	53.6
	Total	27,626	100.0
Birth weight			
	≥2500 g	21,570	77.9
	<2500 g	6102	22.0
	Total	27,672	100.0
Gestational age			
	≥37 weeks	23,425	85.7
	<37 weeks	3892	14.2
	Total	27,317	100.0
Type of pregnancy			
	Single	26,988	97.7
	Twin or more	644	2.3
	Total	27,632	100.0

**Table 4:** Crude association between sex and non-syndromic orofacial cleft types in Brazil between 1999 and 2020.

	Cleft palate		Cleft lip		Cleft lip with palate		p-value
	(n)	(%)	(n)	(%)	(n)	(%)	
Female	4446	39.0	3267	28.7	3667	32.2	<0.001
Male	4989	30.6	5341	32.8	5946	36.5	
Total	9435	34.1	8608	31.1	9613	34.7	

**Figure 1:**
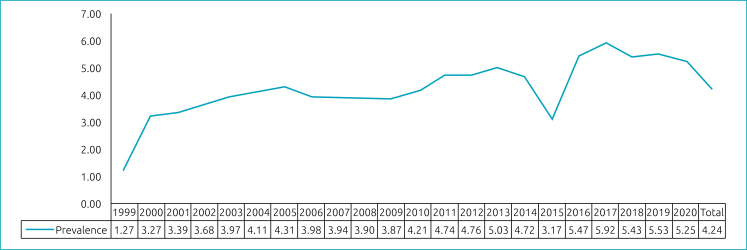
Prevalence of non-syndromic orofacial cleft in Brazil (per 10,000 livebirths), by year.

**Figure 2:**
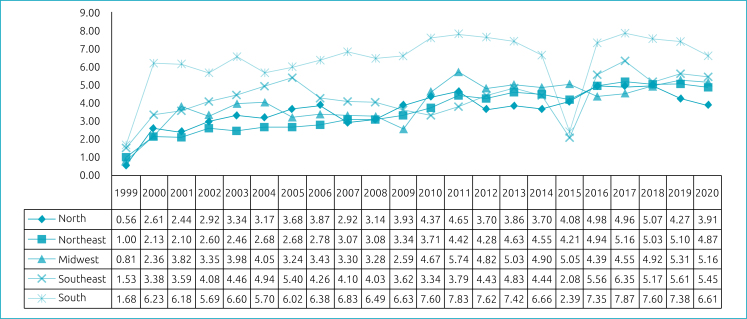
Prevalence of non-syndromic orofacial cleft in Brazil and Regions (per 10,000 livebirths), by year.

At the national level, the prevalence of OC found was 5.16/10,000, and 4.24/10,000 regarding the non-syndromic cases (Figure 1). Also, the prevalence of isolated OC was analyzed at state and federal levels. For the regions, the prevalence was calculated for each year and for the overall period. The South and Southeast regions of Brazil had the highest prevalence — respectively, 6.34/10,000 and 4.26/10,000 —, while the lowest were recorded in the Northeast, with 3.52/10,000, and the North region, with 3.66/10,000 live births. In the Midwest, the prevalence of non-syndromic OC was 4.05/10,000. The state prevalence temporal trend of isolated OC for each year can be found in Figure 2. The prevalence for the period was also calculated for the Federative Units (Table 5).

**Table 5:** Prevalence of non-syndromic orofacial cleft for each Federative Unit from 1999 to 2020 (per 10,000 livebirths).

Federative Unit	Prevalence
Rondônia	5.07
Acre	1.86
Amazonas	3.39
Roraima	4.60
Pará	3.83
Amapá	1.89
Tocantins	3.83
Maranhão	2.13
Piauí	2.38
Ceará	3.95
Rio Grande do Norte	4.97
Paraíba	3.81
Pernambuco	5.47
Alagoas	2.21
Sergipe	4.50
Bahia	2.76
Minas Gerais	3.56
Espírito Santo	3.72
Rio de Janeiro	4.83
São Paulo	4.40
Paraná	6.44
Santa Catarina	6.04
Rio Grande do Sul	6.41
Mato Grosso do Sul	2.87
Mato Grosso	4.90
Goiás	4.57
Distrito Federal	3.08

## DISCUSSION

The results of this study showed that the epidemiological profile of live births with OC in Brazil: individuals with CLP, males, with white race/color, who were born in hospitals with birthweight ≥2500 g and gestational age ≥37 weeks, by cesarean section, with Apgar scores ≥7. Cleft cases were more frequent among mothers who were in their first pregnancy, with single pregnancies, and had seven or more prenatal appointments. These mothers were between 20 and 29 years old, had eight to eleven years of study and were single. The prevalence of NSOC in Brazil was 4.24/10,000. For the Brazilian regions, the South and Southeast had the highest mean prevalence of OC, while the lowest were recorded in the Northeast and North. In relation to the Federative Units, the highest and lowest prevalences of clefts were found, respectively, in Paraná and Acre.

Regarding biological variables, the findings of this study agree with previous ones that also found a higher occurrence of CLP than CP in Brazilian.^18–20^ Also, non-syndromic OC was more frequent in males,^2,21–24^ with a predominance of satisfactory 1^st^ and 5^th^ Apgar scores.^22^ The bivariate analysis found a statistically significant association between sex and OC type, showing a higher occurrence of CP among women and CL/P among men, as in previous studies.^19,20^ For maternal age, there is no consensus in the literature. Although the age group from 20 to 29 years was more predominant in this and other studies,^14,15,23^ the literature also points to a higher occurrence of OC in children whose mothers were of intermediate age, up to 34 years of age,^16,22,23^ and advanced age, from 35 years onwards.^25^ Maternal age differences may have occurred due to possible methodological differences in the studies or possible confounding factors in the analyzed populations. The same occurs for the assessment of ethnicity. Most of those born with any type of non-syndromic OC in Brazil were self-reported as white, in line with previous studies.^24,25^ However, others^15,22,24^ found a higher occurrence of OC in non-white children, who, due to great social inequality, would be distributed in low-income social strata, thus having less access to the health system. These findings may be related to the effect of racial miscegenation in Brazil in the genotype determination, and the lack of standardization in the field of ethnic classification in the Live Birth Declaration.^24,25^ Thus, it is recommended that future studies be carried out to better understand the role of parents’ age and ethnicity in the occurrence of OC.

The epidemiological profile of OC based on maternal and child health care variables was similar to that found by other descriptive studies.^22,24,25^ Among the live births with OC, most of the mothers were in the first, single pregnancy and had had seven or more prenatal consultations. Authors^22^ mention that women, upon receiving the diagnosis of OC in the fetus, decide to carry out more prenatal consultations, or that, when they perform more prenatal appointments, they identify the presence of the malformation and decide to interrupt the pregnancy. It was also found that most children with OC were born by cesarean section, at term and with a weight considered normal. However, this finding may also be associated with the fact that Brazil has a high number of cesarean deliveries, being the second in the top five countries with the highest cesarean section rate worldwide (55.7%).^26^ The fact that the majority of the children born were term reveals normal weight and length gain. In addition, a higher occurrence of normal weight^23,24^ in those born with OC favors their adequacy to extrauterine life, since mortality rates in this weight range are lower.^22^

With regard to socioeconomic and demographic variables, the profile of OC was in agreement with the literature.^14,16,22,23,27^ Despite the distribution of mothers with an average education level among those born with OC in Brazil, it is known that low schooling is a predominant Brazilian characteristic in the profile of neonatal deaths.^28^

Only five^6–8,28,29^ studies were found in the literature regarding the prevalence of OC using SINASC data for the entire country, but none of them covered the entire time period selected for the present analysis. For the period between 1998 and 2002, Rodrigues et al.^6^ found a prevalence of 3.6/10,000 live births. Studies done involving the years between 2009 and 2013 found prevalences ranging from 4.85/10,000 live births^29^ to 5.86/10,000 live births.^28^ The most recent ones^7,8^ found prevalences near 5.1/10,000 live births for the 2005–2017 period. In all of these studies the prevalence of OC showed differences between the Federative Units and the regions. The overall prevalence of OC in Brazil found in this present study is in accordance with what was found in the literature regarding the country;^6–8,28,29^ however, the Brazilian prevalence of this malformation is lower than the global, which is about 1–1.5/1,000 live births.^3^ Some reasons that could explain it are the underestimation of reported cases of OC in the national system, and the misdiagnosis of clefs in some Brazilian regions, especially those with the lowest reports.^30^ Also, SINASC only collects information about live births, so if a child dies during birth or even prior to delivery and she/he has OC this case will not be registered.^29^

Regarding the annual prevalence of OC in Brazil, there has been an increase over the years, with fluctuations in the Brazilian regions, as also found by other authors.^7^ The year 1999 recorded the lowest prevalence rate in the country and in the regions, possibly due to the fact that this was the year in which information on malformations began to be collected and which possibly had the highest occurrence of underreporting, which can be evidenced by the absence of records of OC occurrence in some Brazilian states (Acre, Amapá, Mato Grosso, Mato Grosso do Sul, Pará, Rio Grande do Norte and Roraima). In 2015, there was a decline in the annual prevalence rate of OC in the country, possibly due to a lack of OC records that year in Santa Catarina and São Paulo, which are states with significant numbers of cases recorded over the years. When evaluating the annual behavior of clefts by region, this one-off drop in prevalence in 2015 was also more expressive in the South and Southeast regions, which the aforementioned states are part of.

Analyzing the regions most and least affected by clefts, the results were also consistent with other studies.^6,7,28,29^ This difference between regions can be explained by the greater probability of underreporting of cases in the economically poorer regions,^8^ since, when the OC were evaluated by state, an absence of OC records was found for some years, especially in the Northern states.

Regarding the Federative Units, the prevalence of OC showed disparities, with the highest and lowest found, respectively, in Paraná and Acre. The absence of clefts records in 16 states over the period analyzed may have contributed to these disparities. In addition to the years 1999 and 2015, that do not have records of OC cases in some Federative Units, in 2000 and 2007 there were no OC cases registered in Amapá, in 2001 there were no OC cases in Roraima, and in 2003 there were no records of clefts cases in Acre and Amapá.

Some limitations, typical of the descriptive study model, do not allow this study to establish associations of causal inferences between the analyzed variables and the etiology of these malformations; they only make possible to direct actions (of care, prevention and control) and to formulate causal hypotheses to be tested by analytical studies. The period of time covered in this study was limited to 2020 due to the data availability in the system, which only publishes data regarding the two years previous to the current year. In addition, the main limitations of the present study are related to underreporting and/or errors in filling in the Live Birth Declaration and consequently in SINASC, possibly caused by the turnover and lack of preparation of health professionals to deal with these data. Therefore, maximum completeness should be sought when filling in, and this requires a joint effort from all professionals involved in this process to further increase the credibility of this information.

In Brazil, descriptive studies on OC are usually specific, describing this event in a specific city, state, region or health service. There are few studies with population data in a national context, such as the one reported in the present study. Thus, the results presented here make it possible to direct some prevention and care actions directed at OC and point out the need for analytical studies to assess a possible association between environmental factors and the emergence of these malformations.

## Data Availability

The database that originated the article is available with the corresponding author.
